# A Pilot Study of Neoadjuvant Nivolumab, Ipilimumab, and Intralesional Oncolytic Virotherapy for HER2-negative Breast Cancer

**DOI:** 10.1158/2767-9764.CRC-23-0145

**Published:** 2023-08-23

**Authors:** Vina P. Nguyen, Katie M. Campbell, Theodore S. Nowicki, Nila Elumalai, Egmidio Medina, Ignacio Baselga-Carretero, Maggie L. DiNome, Helena R. Chang, Denise K. Oseguera, Antoni Ribas, John A. Glaspy

**Affiliations:** 1 Department of Medicine, Division of Hematology-Oncology, University of California, Los Angeles, Los Angeles, California.; 2 Jonsson Comprehensive Cancer Center, University of California, Los Angeles, Los Angeles, California.; 3 Parker Institute for Cancer Immunotherapy, San Francisco, California.; 4 Department of Pediatrics, Division of Pediatric Hematology/Oncology, University of California, Los Angeles, Los Angeles, California.; 5 Department of Microbiology, Immunology and Genetics, University of California, Los Angeles, Los Angeles, California.; 6 Department of Surgery, Division of Surgical Oncology, University of California, Los Angeles, Los Angeles, California.; 7 Department of Molecular and Medical Pharmacology, University of California, Los Angeles, Los Angeles, California.

## Abstract

**Purpose:**

Neoadjuvant combination immune checkpoint blockade and intralesional oncolytic virotherapy have the potential to activate antitumor responses in patients with breast cancer.

**Experimental Design:**

Eligibility for this pilot phase I trial included patients with localized HER2-negative breast cancer who received systemic nivolumab and ipilimumab and intratumor talimogene laherparepvec (T-VEC; NCT04185311). The primary objective was to evaluate the safety and adverse event profile of immunotherapy combined with T-VEC in patients with localized, HER2-negative breast cancer.

**Results:**

Six patients were enrolled, 4 having relapses after prior neoadjuvant chemotherapy and 2 who were previously untreated. Toxicities included 1 patient having grade 3 hypotension and type 1 diabetes mellitus, 3 patients with hypothyroidism, and all patients having constitutional symptoms known to be associated with the administration of T-VEC. One patient had a pathologic complete response, 3 patients had pathologic partial responses, 1 showed no significant response, and 1 had disease progression. Biopsies demonstrated increased immune cell infiltration in samples from patients who responded to therapy.

**Conclusions:**

This triple immunotherapy regimen provided responses in patients with advanced or relapsed HER2-negative breast cancer, at the expense of long-term toxicities.

**Significance:**

Systemic immune checkpoint blockade with a programmed death receptor 1 and a CTL antigen-4 blocking antibody, combined with intralesional oncolytic virotherapy, is a chemotherapy-free combination aimed at inducing an antitumor immune response locally and systemic immunity.

## Introduction

Neoadjuvant therapy is a frequently employed strategy for the treatment of patients with high-risk localized breast cancer because it can improve resectability and serve as a benchmark for response to therapy. Moreover, patients with a pathologic complete response (pCR) after neoadjuvant therapy have longer event-free and overall survival (OS; refs. 1–2). However, neoadjuvant chemotherapy alone results in pCR in 50% of patients with triple-negative breast cancers (1). Neoadjuvant immunotherapy could provide long-term response rates by enhancing systemic immunity by reactivating T cells while the primary tumor is still in place (3–4). Mouse models of spontaneous metastatic breast cancers have demonstrated a significantly greater therapeutic activity of neoadjuvant compared with adjuvant immune checkpoint blockade therapy (5). Moreover, immunotherapy in combination with standard neoadjuvant chemotherapy has shown improved response rates in the KEYNOTE-173, KEYNOTE-522, and I-SPY2 clinical trials. The FDA approval in June 2021 of neoadjuvant and adjuvant immunotherapy in combination with chemotherapy in early-stage, high-risk triple-negative breast cancer was based on the KEYNOTE-522 results of improved event-free survival (6–9). Evidence of the benefits of neoadjuvant over adjuvant immune checkpoint blockade therapy has been recently provided in a randomized clinical trial in patients with resectable melanoma (10).

Nivolumab, an anti-PD-1 mAb, and ipilimumab, an anti–CTL-associated antigen 4 (CTLA-4) mAb, are immune checkpoint inhibitors that have shown antitumor activity in several malignancies, including melanoma, renal cell cancer, lung cancer, and hepatocellular cancer (11–14). Anti-PD-1/L1 immunotherapy, alone or in combination with chemotherapy, has been tested for the treatment of patients with metastatic triple-negative breast cancer (15). In the phase II Keynote-086 cohort B, the objective response rate of single-agent pembrolizumab in patients with PD-L1–positive metastatic triple-negative breast cancer was 21.4% (95% confidence interval, 13.9–31.4), with most of the responses being durable (16). In the IMpassion130 randomized clinical trial, which randomized patients to first-line atezolizumab or placebo plus nab-paclitaxel for unresectable, locally advanced, or metastatic triple-negative breast cancer, the improvement OS in the intent-to-treat population was not statistically significant, but the 3-year OS rates in the PD-L1 IC-positive population were 35.8% with atezolizumab plus nab-paclitaxel versus 22.2% with placebo plus nab-paclitaxel (17). Recent efforts have aimed to increase the immunogenicity of breast cancer by combining immunotherapy with other immune-enhancing agents (18–19).

Talimogene laherparepvec (T-VEC) is a genetically engineered herpes simplex virus type 1 (HSV-1), which lacks genes essential for replication in normal tissue and is still sensitive to HSV-1–directed drugs like acyclovir (20). T-VEC is designed to selectively replicate within tumors and produce GMCSF to enhance systemic antitumor immune responses. T-VEC has been approved for use in the treatment of patients with advanced melanoma and has been shown to be safe and effective when combined with checkpoint inhibitors, including anti-PD-1 and anti-CTLA-4 antibodies (21–23).

We reasoned that intralesional injection of T-VEC would induce local immune activation that would prime the activity of combined immune checkpoint blockade with nivolumab and ipilimumab, resulting in tumor responses in patients with locally advanced breast cancer. Therefore, we designed a phase Ib trial with a chemotherapy-free regimen of nivolumab and ipilimumab combined with intralesional T-VEC for neoadjuvant treatment of localized, operable HER2-negative breast cancer. The primary objective was to evaluate the safety and adverse event profile of immunotherapy combined with T-VEC in patients with localized, HER2-negative breast cancer.

## Materials and Methods

### Objectives

The primary objective was to evaluate the safety and adverse event profiles of immunotherapy combined with T-VEC in patients with localized HER2-negative breast cancer. The secondary objective was to assess the potential efficacy and pathologic response to nivolumab, ipilimumab, and T-VEC by measuring the tumor size and by descriptively analyzing the tumor on histopathologic examination after definitive surgery.

### Patient Population

Patients (18 years of age or older) with pathologically proven, palpable, and injectable, localized HER2-negative invasive breast carcinoma with a diameter of 1.0 cm or more were eligible. Despite the low number of patients, enrolled patients represented the diversity of this cancer indication (Supplementary Table S1). Patients were not required to have progressed on prior neoadjuvant chemotherapy, but if they had received prior neoadjuvant chemotherapy, they had to have full recovery from the acute toxic effects with suitable hematologic, renal, and liver function and an Eastern Cooperative Oncology Group performance status of 0 or 1.

### Trial Design and Oversight

Study participants provided voluntary written informed consent approved by the Institutional Review Board (IRB 18-000427) at the University of California, Los Angeles (UCLA). This investigator-initiated trial was conducted in compliance with ethical guidelines under the FDA investigational new drug (IND) #138873, in accordance with the International Council for Harmonization Good Clinical Practice guidelines and the Declaration of Helsinki. The first author, last author, clinical trial coordinator, and UCLA Data Safety and Monitoring Board (DSMB) continuously monitored the safety and data for all patients. The UCLA DSMB met approximately monthly to provide oversight and to serve as the data and safety monitoring committee as part of the UCLA Jonsson Comprehensive Cancer Center Data and Safety Monitoring Plan. The clinical trial was registered with number NCT04185311.

Patients were enrolled at UCLA starting September 2019. The phase Ib study was an open-label, nonrandomized trial that used fixed doses of nivolumab, ipilimumab, and T-VEC, as had previously been FDA approved for other indications, including advanced melanoma. Patients received T-VEC as an intratumoral injection at a concentration of 10^6^ plaque-forming units (PFU) per mL on day 1 and then at a concentration of 10^8^ PFU per mL on days 22 and 36. The volume of T-VEC injected was 1–4 mL, depending on the tumor size, consistent with the current FDA-approved dosing regimen. Nivolumab 240 mg was infused intravenously on days 1, 15, 29, and 43. Ipilimumab 1 mg/kg was infused intravenously on days 1 and 43.

### Safety Evaluation

Patients were continuously monitored for toxic effects and adverse events, which were graded according to the Common Terminology Criteria for Adverse Events version 5.0.

### Response Assessment

The breast tumor was measured using calipers in two dimensions at weekly visits on days 1, 8, 15, 22, 29, 36, and 43. No generally acceptable, well-defined criteria for measuring response after intratumoral oncolytic virotherapy exist; apparent tumor enlargement may be seen that can be associated with new or enlarging central tumor cavities or cysts from tumor-cell death, inflammation related to an influx of immune cells (pseudoprogression), or delayed antitumor immune responses (23–25). Thus, the final response assessment was a pathologic examination at the time of definitive surgical resection of the breast tumor within 45 days after the last treatment.

### Multiplex Immunofluorescence

Multiplex immunofluorescence (mIF) analysis was conducted on baseline and on-treatment biopsies (obtained at the time of tumor resection). Serial sections from the patient tumor samples were deparaffinized and rehydrated with a series of graded ethanol to deionized water on a BOND RX platform (Leica Biosystems). The full details of the antibodies used, antigen retrieval techniques, antibody dilutions, and incubation times are summarized in Supplementary Table S2. Briefly, antigen retrieval was performed in either Ethylenediaminetetraacetic acid (EDTA)-based pH 9 buffer (ER2) or citrate-based pH 6 buffer (ER1). Slides were then serially stained with anti-CD8 clone C8/144B (1:100, DAKO), anti-PD-1 clone NAT105 (1:50; Cell Marque), anti-PD-L1 clone SP142 (1:100; SpringBio), anti-IgG4 clone HP6205 (1:200, Millipore), which served as a marker of nivolumab, or anti-CK7 clone OV-TL (1/200; DAKO), which served as a specific marker for breast carcinoma tissue. The tyramide signal amplification (TSA)-based Opal method was used for mIF staining (Opal Polaris 7‐Color Automation IHC Kit; Akoya Biosciences). Because TSA and 3,3′-diaminobenzidine (DAB) oxidation are both peroxidase‐mediated reactions, the primary antibody conditions and order of staining determined using DAB detection were directly applied to the fluorescent assays. Unlike conventional IHC wherein a chromogenic peroxidase substrate is used for antigen detection, each antibody is paired with an individual Opal fluorophore for visualization. Opal fluorophores were used at a 1:150 dilution. As such, a fluorescent singleplex was performed for each biomarker and compared with the appropriate chromogenic singleplex to assess the staining performance.

Once each target was optimized in the uniplex slides, the Opal multiplexed assay was used to generate multiple staining slides. Primary antibodies were applied to normal human tonsil specimens as controls at optimized concentrations previously determined in uniplex control tissues. Slide Staining was performed consecutively on Leica BOND RX using the same steps as those used in uniplex IF, and the detection for each marker was completed before application of the next antibody. The sequence of antibodies for multiplex staining determined for panel combination was CD8 (opal 480), PDL1 (opal 520), PD1 (opal570), IgG4 (opal 620), CK7 (opal 690), and spectral DAPI (Invitrogen).

All fluorescently labeled slides were scanned on Vectra Polaris (Akoya Biosciences) at 40 × magnification using appropriate exposure times, with 8–10 sections selected from each slide for highest resolution imaging (200x). Sections that did not have any antibodies or fluorescent labeling were used to capture the background tissue autofluorescence. Prior to the analysis, all images were assessed for quality control. The criteria for rejection included poor tissue quality (e.g., folded tissue or missing sections) or staining artifacts (e.g., air bubbles, signal dropout, or inadequate washing). The data from the multispectral camera were analyzed using the InForm imaging software (Akoya Biosciences) and Halo (Indica Labs).

### Exome and RNA Sequencing

DNA and RNA were extracted from formalin-fixed, paraffin-embedded (FFPE) tumor biopsies using the Covaris truXTRAC FFPE total NA Plus (Column) kit and the Covaris M220 focused ultrasonicator (Covaris), respectively, according to the manufacturer's protocol with several modifications. Two 15 μm scrolls were sectioned from FFPE blocks by the UCLA Translational Pathology Core Laboratory, and each scroll was individually processed for paraffin emulsification, proteinase K, and centrifugation. RNA-containing supernatants or DNA-containing tissue pellets from two individual scrolls were combined in their respective columns for washing and elution. RNA quality and quantity were evaluated using the Agilent Bioanalyzer RNA Pico kit (Agilent Technologies), and DNA quality and quantity were assessed using the Agilent Tapestation 4200 and Genomic DNA screen tape (Agilent Technologies).

RNA (RNA-seq) and whole-exome sequencing (WES) library preparation were automated on the Agilent BRAVO Platform (Agilent Technologies). WES libraries were prepared using the Agilent SureSelect XT HS2 DNA library preparation system, with hybridization capture by the Agilent SureSelect v7 Exome with the Agilent OneSeq CNV Backbone panel according to the manufacturer's protocol. RNA-seq libraries were prepared using the Agilent SureSelect XT HS2 RNA library preparation system, with hybridized capture of cDNA using the Agilent SureSelect v7 Exome reagent. Pools (2 nmol/L) of either WES or RNA-seq libraries were sequenced by the UCLA Technology Center for Genomics and Bioinformatics core laboratory on the Illumina Novaseq (S4 flow cell, 2 × 150 bp reads).

### Sequencing Data Analysis

WES data were preprocessed according to GATK best practices (26) and aligned to the human reference genome (GRCh38) using BWA-MEM v0.7.15 (arXiv 2013;1303.3997). Mutations were detected by comparing patient-matched tumors and normal WES using Mutect2 (bioRxiv 2019:861054), Varscan2, Strelka, and SomaticSniper (27–29). Single-nucleotide variants (SNV) were further annotated to remove false-positive artifacts using DeepSVR (30). Neighboring SNVs were evaluated to deduplicate multi-nucleotide variants (MNV) by detecting a minimum of three shared reads corresponding to sequential SNV calls. The final set of SNVs and small insertions and deletions (indel) was filtered to those identified by at least two variant callers, SNVs that passed DeepSVR somatic annotation, and individual SNVs corresponding to MNVs were removed. SNVs and indels were annotated by the Ensembl Variant Effect Predictor (VEP), using the Ensembl annotation database v94, and “nonsilent” mutations were those with either “HIGH” or “MODERATE” impacts on protein structure or function, as defined by Ensembl VEP. Tumor mutational burden (TMB) was quantified by normalizing the number of nonsilent mutations (with variant allele frequencies greater than 5%) to the size of the genome (in megabases) that had at least 50X sequencing coverage. TMB was summarized at the patient level using the baseline biopsy; if the baseline biopsy was not available, the surgical specimen was used.

T-VEC was quantified in the WES data by the detection of the cytomegalovirus (CMV) promoter sequence, encoded in the viral vector, upstream of *CSF2*. Read alignments spanning the translation start site of *CSF2* (GRCh38, chr5:132,073,823-132,073,824) were extracted from the GRCh38-aligned WES data, and the 100 bp upstream of the translation start site were compared with the CMV promoter sequence (GGGAGGTCTATATAAGCAGAGCTCTCTGGCTAACTAGAGAACCCACTGCTTACTGGCTTATCGAAATTAATACGACTCACTATAGGGAGACCCAAGCTT). All reads were manually reviewed, and the number of reads that passed the manual review for the inclusion of the CMV promoter and *CSF2*-spanning reads was used to quantify the presence of T-VEC for each sample.

RNA-seq data were aligned to the human reference genome (GRCh38) using HISAT2 (31), and gene expression was quantified using Stringtie (32). Immune cell type gene expression scores were quantified by MCPcounter using the input gene expression Fragments Per Kilobase of transcript per Million mapped reads (FPKM) values (33).

### Statistical Analysis

The sample size was initially intended to be 20 subjects, but due to slow accrual during the COVID-19 pandemic, the study was closed early after 6 subjects were enrolled. All patients who received ≥1 dose of the triple therapy combination were included in the intention-to-treat population and evaluated for safety and antitumor activity. Adverse events were summarized according to frequency, grade, and relationship with each trial drug. Descriptive statistics were tabulated for tumor size. Given the exploratory nature of this study, no adjustments for multiple comparisons were made, and no formal comparisons of dose levels were performed for any endpoints.

### Data Availability

Raw sequencing data derived from cell line and normal peripheral blood mononuclear cell control samples collected under UCLA IRB approval no. 18-000427 deposited to the database of Genotypes and Phenotypes (dbGaP) under accession number phs003316.v1 (https://www.ncbi.nlm.nih.gov/projects/gap/cgi-bin/study.cgi?study_id=phs003316.v1.p1).

## Results

### Patient Characteristics and Treatment

Between September 2019 and April 2021, 6 patients (Table 1; Supplementary Table S3) were enrolled and completed the trial (age range, 40–72 years). Four patients had triple-negative breast cancer and 2 had hormone receptor-positive, *HER2* nonamplified breast cancer. The median tumor size was 3.35 cm (range, 1.5–6.5 cm). Four patients had clinically positive nodes at baseline (2 were confirmed by biopsy). Four patients had received prior chemotherapy. All patients had been recommended to receive neoadjuvant chemotherapy, but 2 (33%) declined for personal reasons. Three patients had recurrent breast cancers. Patients received three intratumoral injections of T-VEC, starting with a sensitizing dose (1–4 × 10^6^ PFU per mL depending on tumor size) on day 1, followed by full doses of 1–4 × 10^8^ PFU per mL on days 22 and 36, nivolumab 240 mg intravenously on days 1, 15, 29, and 43, and ipilimumab at 1 mg/kg intravenously on days 1 and 43.

**TABLE 1:** Baseline characteristics

Characteristic	All patients (*n* = 6)
Median age, years (range)	55 (40–72)
Hormone receptor positive, *n* (%)	2 (33%)
Triple negative, *n* (%)	4 (67%)
Median initial tumor size in largest dimension, cm (range)	3.35 (1.5–6.5)
Prior chemotherapy, *n* (%)	4 (67%)
Recurrence, *n* (%)	3 (50%)
Positive nodes, *n* (%)	4 (67%)
Stage I, *n* (%)	1 (16%)
Stage II, *n* (%)	2 (33%)
Stage III, *n* (%)	3 (50%)

### Safety Outcomes

One patient had two grade 3 adverse events (Table 2). This patient first developed syncope due to hypovolemic hypotension secondary to acute diarrhea within hours of receiving the third T-VEC injection, which was complicated by grade 1 acute kidney injury that resolved with intravenous hydration. There was also an initial suspicion of adrenal insufficiency that could not be confirmed upon further testing. Because of this possible immune-related adverse event, the patient did not receive the last treatment infusion during the trial. The same patient later developed diabetic ketoacidosis secondary to new-onset type 1 diabetes mellitus, requiring hospitalization a month after the last treatment. This patient also developed hypothyroidism earlier during the treatment course. Three patients developed grade 1 hyperthyroidism that gradually transitioned to hypothyroidism, requiring thyroid hormone replacement therapy within weeks (Table 2). The patients were asymptomatic at the time of diagnosis of thyroid dysfunctions. All other adverse events were grade 1–2, the most common being chills, fatigue, and malaise in 5 patients. The next most common adverse events were fever, nausea, injection site pain, and myalgia in 4 patients (Table 2). These are known side effects of T-VECs.

**TABLE 2:** Adverse events (total *n* = 6)

Event	Grade 1	Grade 2	Grade 3	Grade 4
Any adverse event	6 (100%)	3 (50%)	1 (17%)	0
*Treatment-related adverse events*				
Chills	5 (83%)	0	0	0
Fatigue	5 (83%)	0	0	0
Malaise	5 (83%)	0	0	0
Fever	4 (67%)	0	0	0
Nausea	4 (67%)	0	0	0
Injection site pain	4 (67%)	0	0	0
Myalgia	4 (67%)	0	0	0
Vomiting	2 (33%)	0	0	0
Diarrhea	2 (33%)	0	0	0
Neutropenia	1 (17%)	1 (17%)	0	0
Rash	2 (33%)	0	0	0
Pruritus	2 (33%)	0	0	0
Syncope	0	0	1 (17%)	0
AKI	1 (17%)	0	0	0
*Immune-related adverse events*				0
Hypothyroidism	0	3 (50%)	0	0
Hyperthyroidism	3 (50%)	0	0	0
Transaminitis	2 (33%)	0	0	0
Adrenal insufficiency	1 (17%)	0	0	0
Type 1 diabetes mellitus	0	0	1 (17%)	0

NOTE: All treatment-related adverse events occurred during the trial period or within 30 days of the trial period (within 90 days for serious events). The severity of the adverse events was graded according to the Common Terminology Criteria for Adverse Events (version 5.0) of the NCI. Patients may have experienced more than one adverse event.

Abbreviation: AKI, acute kidney injury.

### Pathologic Outcomes

All patients underwent definitive surgery within 1 month of the end-of-study drug administration. Four patients underwent mastectomy with either axillary lymph node dissection or sentinel lymph node biopsy, depending on the initial nodal status. Two patients underwent lumpectomies and sentinel lymph node biopsies due to patient preference. Pathologic analysis of the surgical specimens demonstrated that 1 patient had a pCR, with no residual invasive carcinoma and zero of the three sentinel nodes involved with carcinoma. Three patients had residual cancer burden (RCB) class II. One patient had no significant response (RCB class III), and one patient had disease progression at the time of surgery (Table 3).

**TABLE 3:** Tumor sizes, response to therapy, and follow-up events

Case #	Initial tumor size in cm	Final tumor size in cm	Autoimmune toxicities	Pathologic response to therapy	Follow-up therapies	Posttreatment relapse	Recurrence-free survival in months (location of recurrence)	Current status	Overall survival in months
1	6.5	8.4	Hypothyroidism	Progression	Nivolumab and eribulin	Had continued progression on therapy	0	Deceased	6
2	3.4	4	None	Partial response, RCB-II (ypT2 pN0)	Capecitabine	No	29+	Alive	29+
3	1.5	0.8	Hypothyroidism	Pathological complete response	Capecitabine	No	33+	Alive	33+
4	3	2.2	None	Partial response, RCB-III (ypT2 pN1a)	ddAC and capecitabine, gencitabine and carbotaxol, tucatinib/ trastuzumab, sacituzumab	Yes	17 (brain metastases)	Deceased	23
5	3.3	2.3	None	Partial response, RCB-II (rypT1a pN1a)	none	Yes	13 (nodal metastasis)	Alive	19+
6	6.1	3.9	Hypothyroidism, rash, T1DM	Partial response, RCB-II (ypT1a pN0)	none	No	17+	Alive	17+

### Follow-up

**FIGURE 1:**
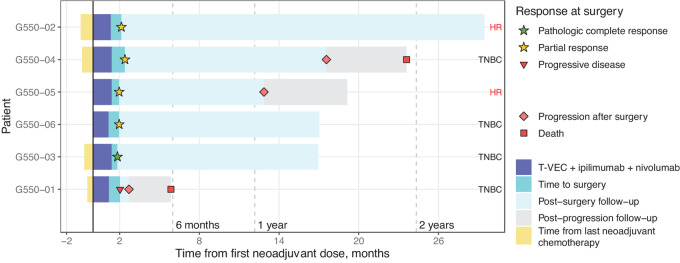
Treatment response duration and follow-up of patients enrolled in the phase I trial. Swimmer lane plot of time on study, noting the period from prior neoadjuvant chemotherapy to study start in the 4 patients who had prior treatment, the period of time on study drugs, the pathologic response assessment, timing of surgery, and follow-up period with events of progression or death.

The median follow-up time was 23 months (range, 19–33 months for patients who were alive; Fig. 1; Table 3). Four patients were alive and 3 were in remission at the end of follow-up. One patient (case #1) with triple-negative breast cancer and concomitant sickle cell disease died of disease progression less than 6 months after starting the trial. Two patients (cases #2 and #3) received adjuvant chemotherapy with capecitabine for 6 months and continued without relapse at 29+ and 33+ months. One patient (case #4) with triple-negative breast cancer who had received neoadjuvant chemotherapy with carboplatin and docetaxel prior to enrollment in the trial had pT2 pN1a disease at the time of surgery (RCB-III), went on to receive adjuvant dose-dense cyclophosphamide and doxorubicin for four cycles followed by capecitabine for 6 months postoperatively. This patient subsequently developed brain metastases at 17 months, and died of disease progression 23 months after initiation of clinical trial participation. Two patients (cases #5 and #6) with hormone receptor–positive breast cancer declined the recommended adjuvant hormone therapy: one progressed at 11 months and was alive at 19+ months, and the other continued to be recurrence free at 17+ months of follow-up (Fig. 1).

### Tumor Biopsy Analyses

**FIGURE 2:**
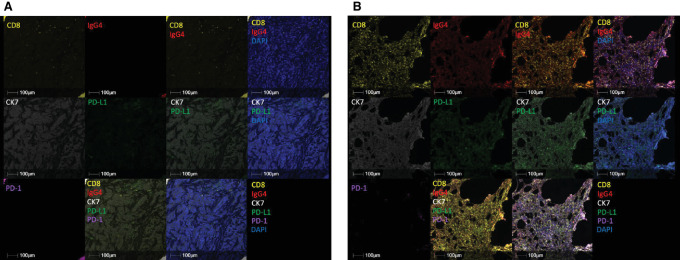
Correlative analysis of tumor specimens. Representative baseline biopsy (**A**) and on-treatment surgical resection (**B**) of a patient who responded to treatment. Color scheme, Yellow: CD8, Red: IgG4, White: CK7, Green: PD-L1, Magenta: PD-1, Blue: DAPI. Baseline biopsy demonstrates PD-L1 signal and minimal CD8 infiltration, with no detectable PD-1 or IgG4 signal, while on-treatment biopsy demonstrated diffuse CD8 infiltration colocalized with strong IgG4 signal (double positive = orange), along with minimal PD-1 signal, consistent with the visualization of the IgG4 antibody nivolumab saturating the PD-1 receptor on infiltrating T cells.

mIF was used to analyze changes in the tumor microenvironment (TME) induced by the combined immunotherapy. Samples were available from all 6 patients (five baseline, six surgery; five paired; Supplementary Table S4) and were stained to detect cytokeratin 7 to determine breast cancer cells, PD-L1 expression, and changes in CD8 effector T cells recruited by the combined immunotherapy. Samples were also stained with anti-IgG4, the clonotype of nivolumab, and anti-PD-1 using an antibody cross-reactive to nivolumab, which provided direct visualization of PD-1 receptor target saturation by nivolumab in the tumor. Baseline biopsies demonstrated PD-L1 signal and minimal CD8 infiltration, with no detectable PD-1 or IgG4 signals. The on-treatment resection tissue demonstrated diffuse CD8 infiltration colocalized with a strong IgG4 signal, along with minimal PD-1 signal, demonstrating PD-1 receptor target engagement on cytotoxic CD8^+^ T cells by nivolumab in the tumor (Fig. 2A and B). Conversely, the on-treatment resection tissue from a patient with no response to treatment demonstrated only minor CD8 T-cell infiltration within viable tumor tissue, with no detectable PD-1, PD-L1, or IgG4 signal present (Supplementary Fig. S1).

**FIGURE 3:**
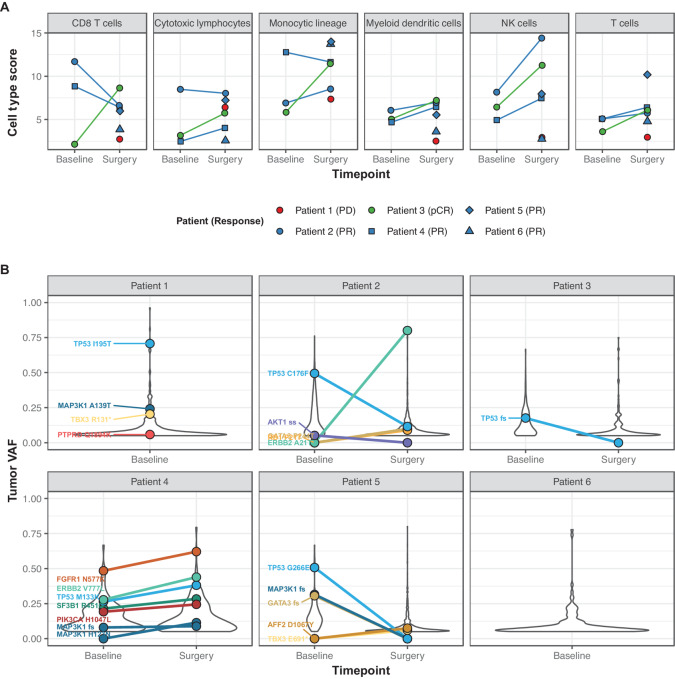
RNA-seq and DNA sequencing analyses of biopsies. **A,** MCPcounter was used to summarize gene expression scores for immune cell populations from bulk RNA-seq expression data across cell types. Each point represents one sample, and paired baseline/surgery samples are connected by a line (three paired biopsies); individual patients and their responses are indicated by color and point shape. **B,** Summary of somatic driver mutations by variant allele fraction (VAF). Variants detected at both timepoints are connected by a line (four paired biopsies), and mutations in known genetic drivers of breast cancer are indicated with colored labels.

Tumor samples were assessed using RNA-seq to further investigate the TME. Samples were available from all 6 patients (three baseline, six surgery, and three paired). Immune cell types in the resected samples compared with baseline biopsies, assessed by RNA-seq data deconvolution, showed distinct increases in gene expression associated with CD8^+^ T cells, cytotoxic lymphocytes, monocytes, and natural killer (NK) cells over the course of treatment in patients with pCR (Fig. 3A; Supplementary Fig. S2). Samples from patients with pathologic partial response (pPR) or stable disease exhibited higher expression of these cell types, often in both baseline and surgery specimens. When samples were queried for the presence of T-VEC by analyzing the WES data to detect the CMV promoter sequence present in the T-VEC viral vector upstream of the human *CSF2* gene encoding GMCSF, the surgical sample of only one patient with partial response (case #2; Supplementary Fig. S3) showed a signal of the virus, suggesting that the injected viral vector was quickly cleared by an antiviral response in most cases. The surgical sample from the patient with progressive disease appeared to be an outlier across most immune cell type analyses compared with the other surgical samples, with the lowest signals of genes associated with T cells, monocytes, myeloid dendritic cells, neutrophils, and NK cells (Supplementary Fig. S2).

WES of tumor specimens was used to investigate the genetic drivers and potential tumor clonal dynamics with respect to therapy. Known genetic drivers of breast cancer were detected in biopsies of 5 patients, including *TP53* mutations in samples from 5 of 6 patients, hotspot mutations in *FGFR1* (*n* = 1), *ERBB2* (*n* = 1) and *PIK3CA* (*n* = 1), and mutations in *TBX3* (*n* = 2) and *MAP3K1* (*n* = 3; Fig. 3B). Nonsilent TMB ranged from 3.8 to 13.4 Mut/Mb (median, 6.0 Mut/Mb). WES from paired baseline and surgery samples were available for 4 patients. One patient had a *TP53* frameshift deletion detected at baseline and not at the time of surgery, consistent with the pCR in this patient. Driver mutations in 2 patients with pPR (cases #2 and #5) showed reduced presence at the time of surgery. The samples from the patient with stable disease (case #4) showed maintenance of canonical drivers over the course of the therapy (Fig. 3B).

## Discussion

Neoadjuvant immunotherapy combining systemic immune checkpoint blockade with intralesional oncolytic virotherapy could induce a local antitumor response in breast cancer and result in long-lasting systemic immune responses, preventing the future development of metastatic disease. In this phase Ib clinical trial, the combination of nivolumab and reduced-dose ipilimumab with intralesional T-VEC was found to have local antitumor activity in patients with locally advanced HER2-negative breast cancer. Common adverse events consistent with the toxicity profile of T-VEC, including flu-like symptoms within the first few days of T-VEC administration, were noted in all patients. Despite using reduced-dose ipilimumab, this triple combination resulted in irreversible long-term toxicities in 1 patient with type 1 diabetes mellitus and 3 patients with hypothyroidism. Hypothyroidism with nivolumab and ipilimumab is a significant problem (34–36). As our sample size is very small, the exact rate of hypothyroidism toxicity from this three-agent combination immunotherapy is not yet known. Careful monitoring of immune-related adverse events will be needed in any future trials testing the combination of nivolumab, ipilimumab, and T-VEC, in particular because the endocrine toxicities were all long term and not reversible.

The current study showed antitumor activity, with 1 patient having a pCR and 3 with evidence of tumor regression without complete response. The 2 patients without a pathologic response to therapy had aggressive disease biology at baseline; both had triple-negative breast cancer and positive nodes at diagnosis, and both had already received neoadjuvant chemotherapy without response. Of note, 4 of the 6 patients enrolled in the trial had clinically residual disease after neoadjuvant chemotherapy, which suggests a more aggressive disease. Of these 4 patients, 1 obtained a pCR; this patient had triple-negative breast cancer with the smallest tumor at baseline (1.5 cm) and was node negative clinically at baseline. This patient had enrolled on this trial because she was diagnosed with a second breast cancer at the young age of 47, and had residual disease after neoadjuvant chemotherapy. While pCR is not as robust of an outcome as OS, pCR has been shown to correlate with disease-free survival in prior breast cancer trials (1–2). In our clinical trial, patients received both systemic and local therapy, so it is possible that pCR may also correlate with improved local and systemic outcomes as in prior trials. Moreover, in this trial, the 2 patients with the worst responses at the time of surgery had the shortest OS, less than 2 years after enrolling on the trial.

The recent phase II BELLINI clinical trial evaluating nivolumab and ipilimumab in early-stage triple-negative breast cancer reported a partial radiological response in 7 of 31 (23%) patients after 4 weeks (35). In comparison, the phase II NIMBUS trial evaluating nivolumab and ipilimumab in patients with metastatic HER2-negative breast cancer with high TMB showed partial response in 4 of 30 (13%) patients, although patients with higher TMB (14 Mut/Mb or higher) seemed to have better response rates (60%; ref. 36).

The major limitation of this trial was the limited number of patients enrolled and the heterogeneity of the patients. The eligibility criteria had been deliberately broad (i.e., any localized breast cancer that was palpable, >1 cm, and HER2-negative) because it was a pilot clinical trial, and because of initial concerns about enrollment as well. Furthermore, the trial was opened in September 2019, immediately prior to the COVID-19 pandemic, which also limited enrollment. The trial was closed in April 2021 due to slow enrollment.

While this study was limited to 6 patients, the tumor biopsies revealed several intriguing patterns. Both mIF and bulk RNA-seq analyses revealed increased signals associated with CD8^+^ T cells and cytotoxic lymphocytes in after neoadjuvant therapy resection samples from patients with pathologic responses. These patterns were higher in samples from patients with partial responses to neoadjuvant treatment than in the sample from a patient with a complete response, probably reflecting that the samples with pathologic partial response were closer to the peak of an antitumor immune response, while the one with pCR had started tissue healing. In contrast, the resection sample from the patient who progressed on therapy was a distinct outlier when assessing the TME, without evidence of an immune infiltrate. These patterns are consistent with previous studies describing the mechanistic role of CD8^+^ T cells in immune checkpoint blockade therapies as well as the putative recruitment of tumor-reactive T cells to the tumor site (37).

In conclusion, the novel combination of T-VEC with nivolumab and ipilimumab as a neoadjuvant treatment for HER2-negative early-stage breast cancer showed antitumor activity at the cost of irreversible long-term toxicities, with type 1 diabetes mellitus in 1 patient and hypothyroidism in 3 patients. Further studies are needed to determine whether there will be any role for T-VEC in the treatment of breast cancer.

## Supplementary Material

Supplementary Figure S1 Surgical specimens after neoadjuvant therapy from patients who did not respond to treatment. Color scheme: Yellow: CD8, Red: IgG4, White: CK7, Green: PD-L1, Magenta: PD-1, Blue: DAPI. Staining demonstrates minor CD8 T-cell infiltration within viable tumor tissue, with no detectable PD-1, PD-L1, or IgG4 signal present.

Supplementary Figure S2 Immune cell deconvolution of bulk RNA-seq data. MCPcounter was used to summarize gene expression scores for immune cell populations from bulk RNA-seq expression data across ten cell types. Each point represents one sample, and paired baseline/surgery samples are connected by a line. Individual patients and their responses are indicated by color and point shape.

Supplementary Figure S3 Detection of talimogene laherparepvec (T-VEC) vector reads in case #2. This screenshot from the integrated genomics viewer shows reads aligning to CSF2 (GM-CSF) from the peripheral blood, baseline, and end-of-treatment (EOT) whole exome sequencing (WES) data from the surgical specimen of case #2. The short-clipped ends of the reads at the 5’ end of CSF2 contained the T-VEC cytomegalovirus (CMV) promoter sequence, and reads spanned the exon-exon junctions of the CSF2 transcript. This pattern was not observed in the surgical (EOT) specimens from the other patients or in baseline samples.

Supplementary Table S1 Representativeness of Study Participants

Supplementary Table S2 Summary of antibodies, antigen retrieval conditions, incubation conditions, and opal dye labeling for multiplex immunofluorescence experiments

Supplementary Table S3 Individual details of baseline patient characteristics

Supplementary Table S4 Biopsy samples for analyses
