# Supplementary material for: A Pilot Study of Neoadjuvant Nivolumab, Ipilimumab, and Intralesional Oncolytic Virotherapy for HER2-negative Breast Cancer
Source: Cancer Res Commun. 2023 Aug 23;3(8):1628–37. doi: 10.1158/2767-9764.CRC-23-0145 (PMC10445661; doi:10.1158/2767-9764.CRC-23-0145)
Supplement: Supplementary Figure S1 — Surgical specimens after neoadjuvant therapy from patients who did not respond to treatment. Color scheme: Yellow: CD8, Red: IgG4, White: CK7, Green: PD-L1, Magenta: PD-1, Blue: DAPI. Staining demonstrates minor CD8 T-cell infiltration within viable tumor tissue, with no detectable PD-1, PD-L1, or IgG4 signal present. [file crc-23-0145-s01.pptx]

## Slide 1
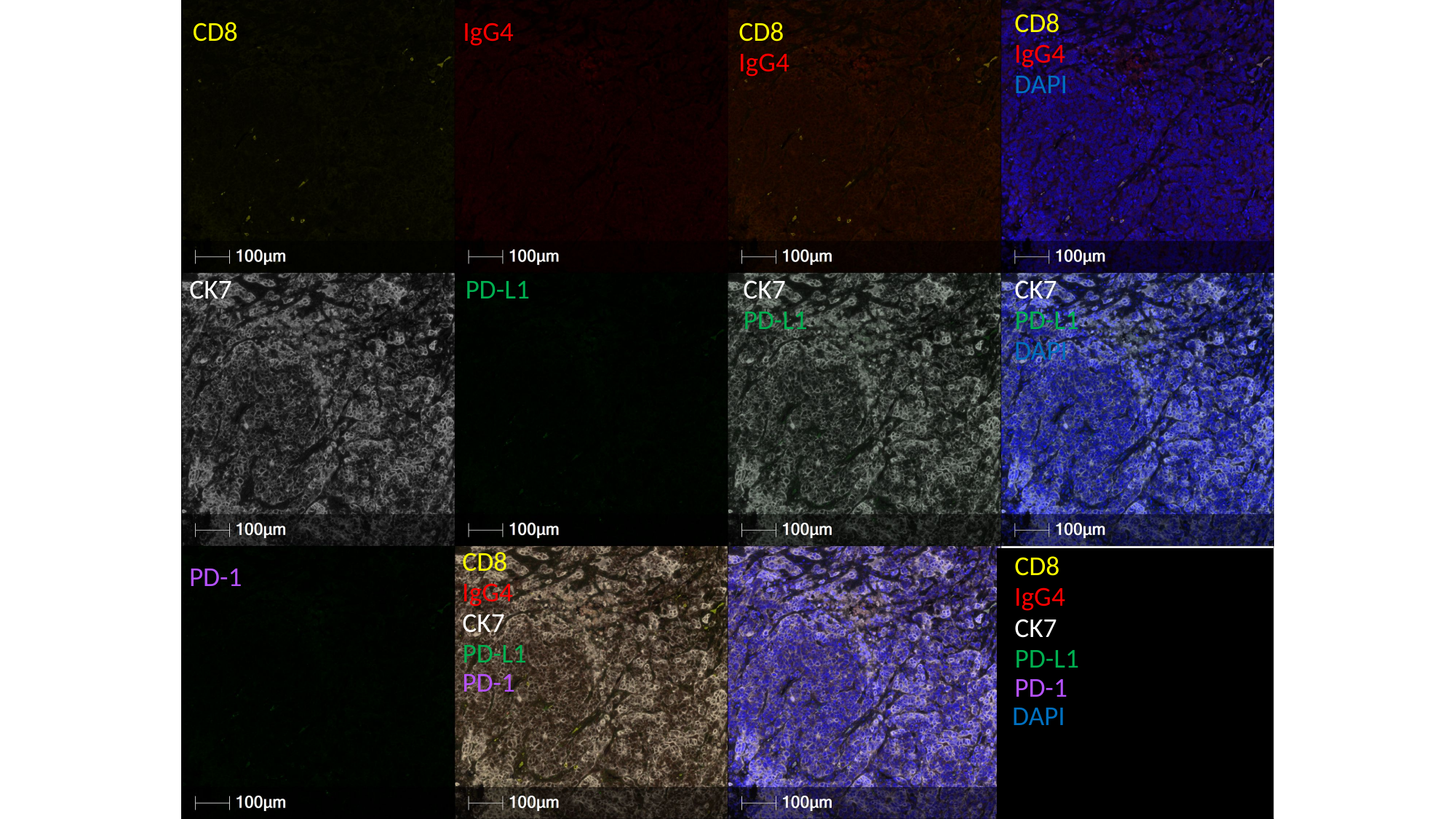

CD8
CD8
IgG4
CD8
IgG4
IgG4
DAPI
CK7
PD-L1
CK7
CK7
PD-L1
PD-L1
DAPI
CD8
CD8
PD-1
IgG4
IgG4
CK7
CK7
PD-L1
PD-L1
PD-1
PD-1
DAPI

## Slide 2
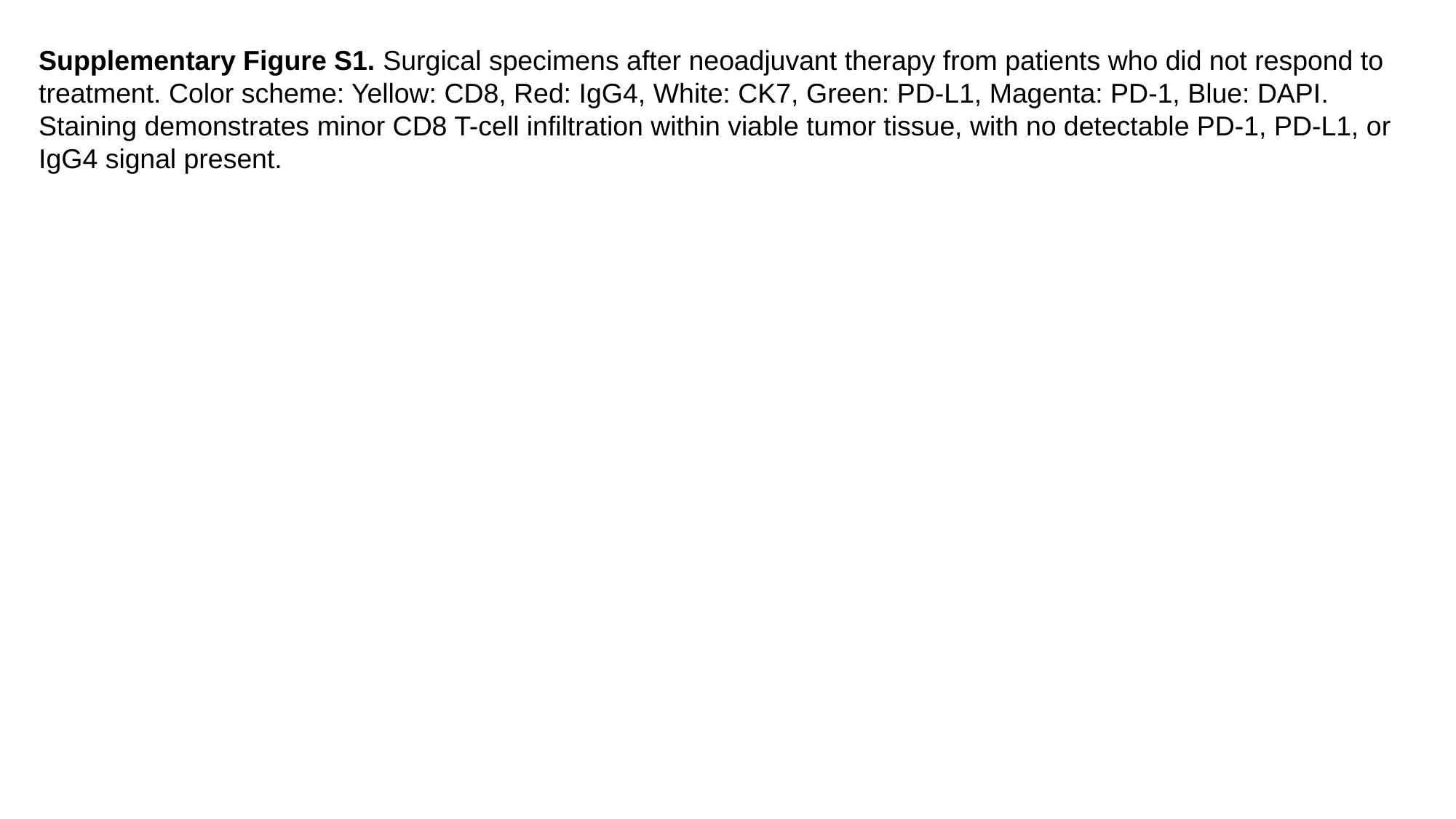

Supplementary Figure S1. Surgical specimens after neoadjuvant therapy from patients who did not respond to treatment. Color scheme: Yellow: CD8, Red: IgG4, White: CK7, Green: PD-L1, Magenta: PD-1, Blue: DAPI. Staining demonstrates minor CD8 T-cell infiltration within viable tumor tissue, with no detectable PD-1, PD-L1, or IgG4 signal present.
