# Supplementary material for: A Pilot Study of Neoadjuvant Nivolumab, Ipilimumab, and Intralesional Oncolytic Virotherapy for HER2-negative Breast Cancer
Source: Cancer Res Commun. 2023 Aug 23;3(8):1628–37. doi: 10.1158/2767-9764.CRC-23-0145 (PMC10445661; doi:10.1158/2767-9764.CRC-23-0145)
Supplement: Supplementary Figure S2 — Immune cell deconvolution of bulk RNA-seq data. MCPcounter was used to summarize gene expression scores for immune cell populations from bulk RNA-seq expression data across ten cell types. Each point represents one sample, and paired baseline/surgery samples are connected by a line. Individual patients and their responses are indicated by color and point shape. [file crc-23-0145-s02.pptx]

## Slide 1
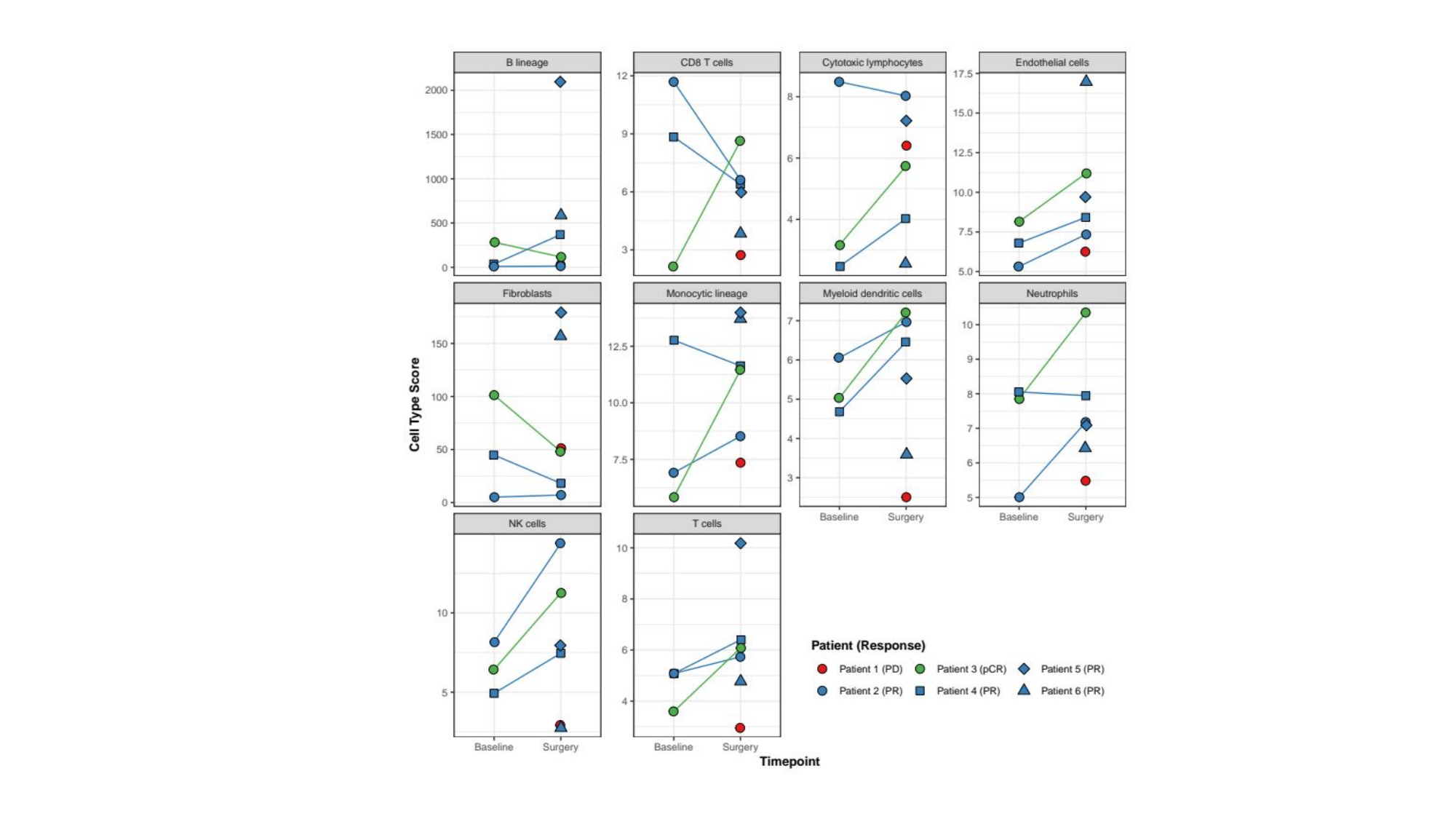

## Slide 2
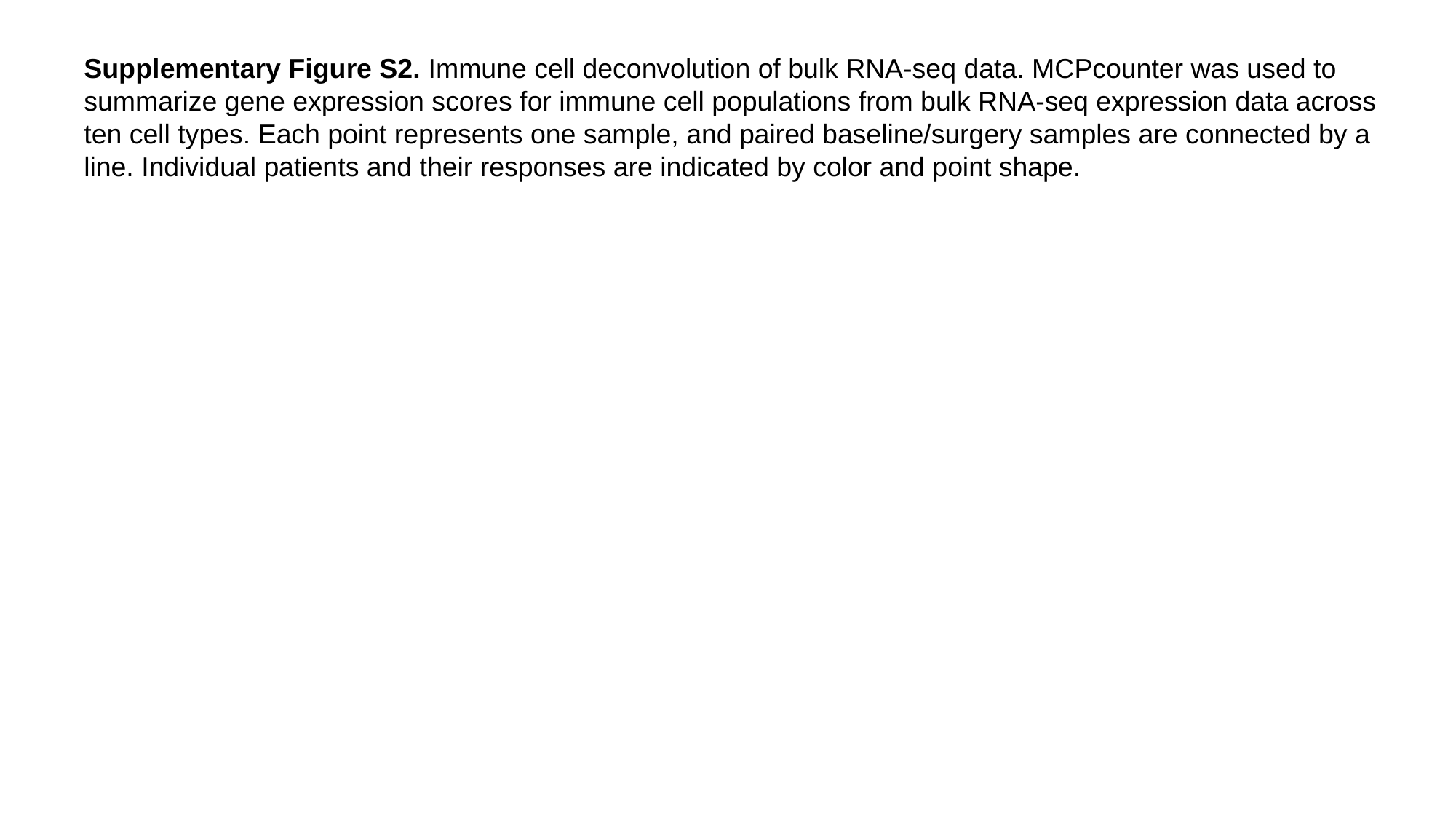

Supplementary Figure S2. Immune cell deconvolution of bulk RNA-seq data. MCPcounter was used to summarize gene expression scores for immune cell populations from bulk RNA-seq expression data across ten cell types. Each point represents one sample, and paired baseline/surgery samples are connected by a line. Individual patients and their responses are indicated by color and point shape.
