# Supplementary material for: A Pilot Study of Neoadjuvant Nivolumab, Ipilimumab, and Intralesional Oncolytic Virotherapy for HER2-negative Breast Cancer
Source: Cancer Res Commun. 2023 Aug 23;3(8):1628–37. doi: 10.1158/2767-9764.CRC-23-0145 (PMC10445661; doi:10.1158/2767-9764.CRC-23-0145)
Supplement: Supplementary Figure S3 — Detection of talimogene laherparepvec (T-VEC) vector reads in case #2. This screenshot from the integrated genomics viewer shows reads aligning to CSF2 (GM-CSF) from the peripheral blood, baseline, and end-of-treatment (EOT) whole exome sequencing (WES) data from the surgical specimen of case #2. The short-clipped ends of the reads at the 5’ end of CSF2 contained the T-VEC cytomegalovirus (CMV) promoter sequence, and reads spanned the exon-exon junctions of the CSF2 transcript. This pattern was not observed in the surgical (EOT) specimens from the other patients or in baseline samples. [file crc-23-0145-s03.pptx]

## Slide 1
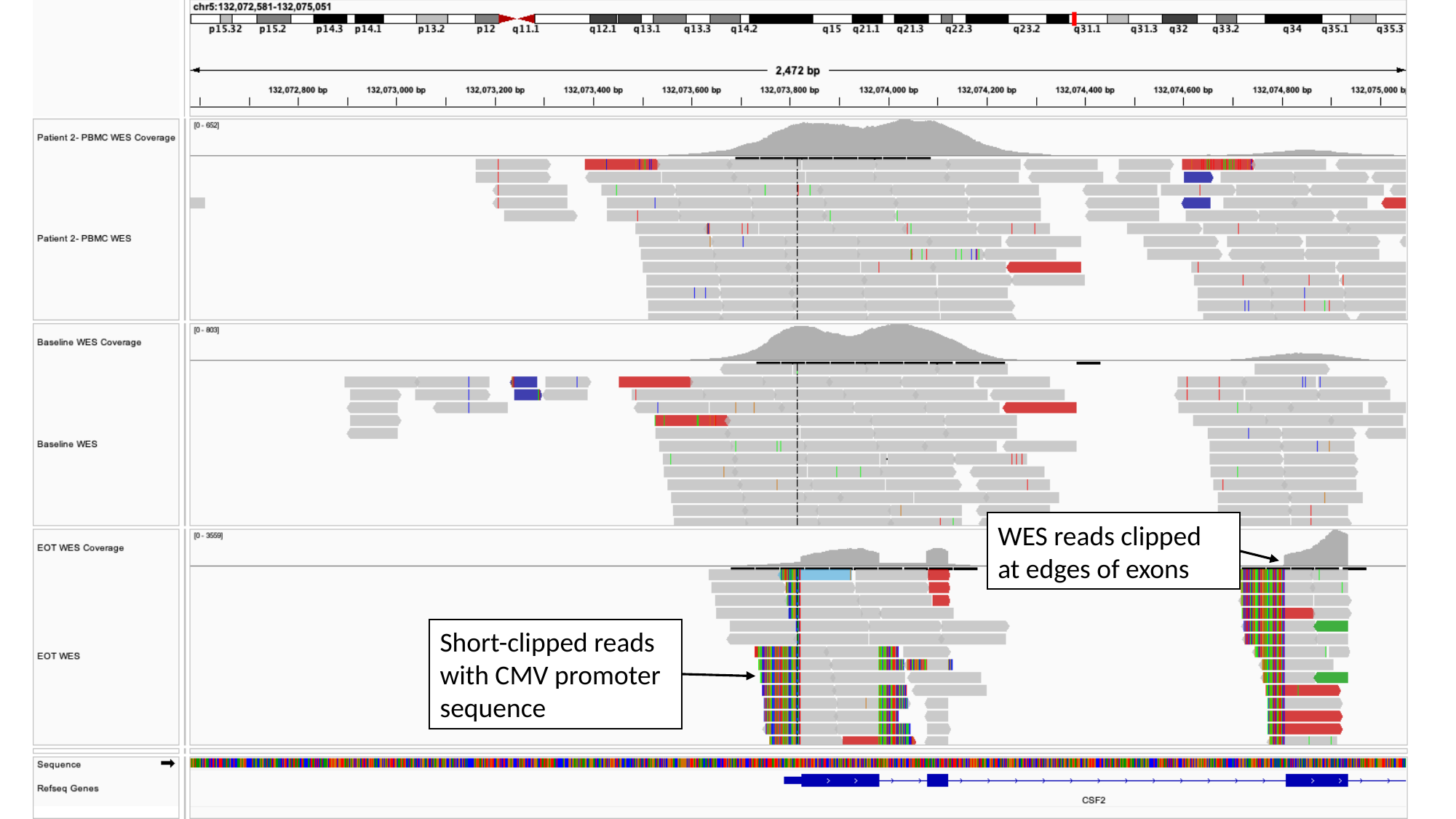

WES reads clipped at edges of exons
Short-clipped reads with CMV promoter sequence

## Slide 2
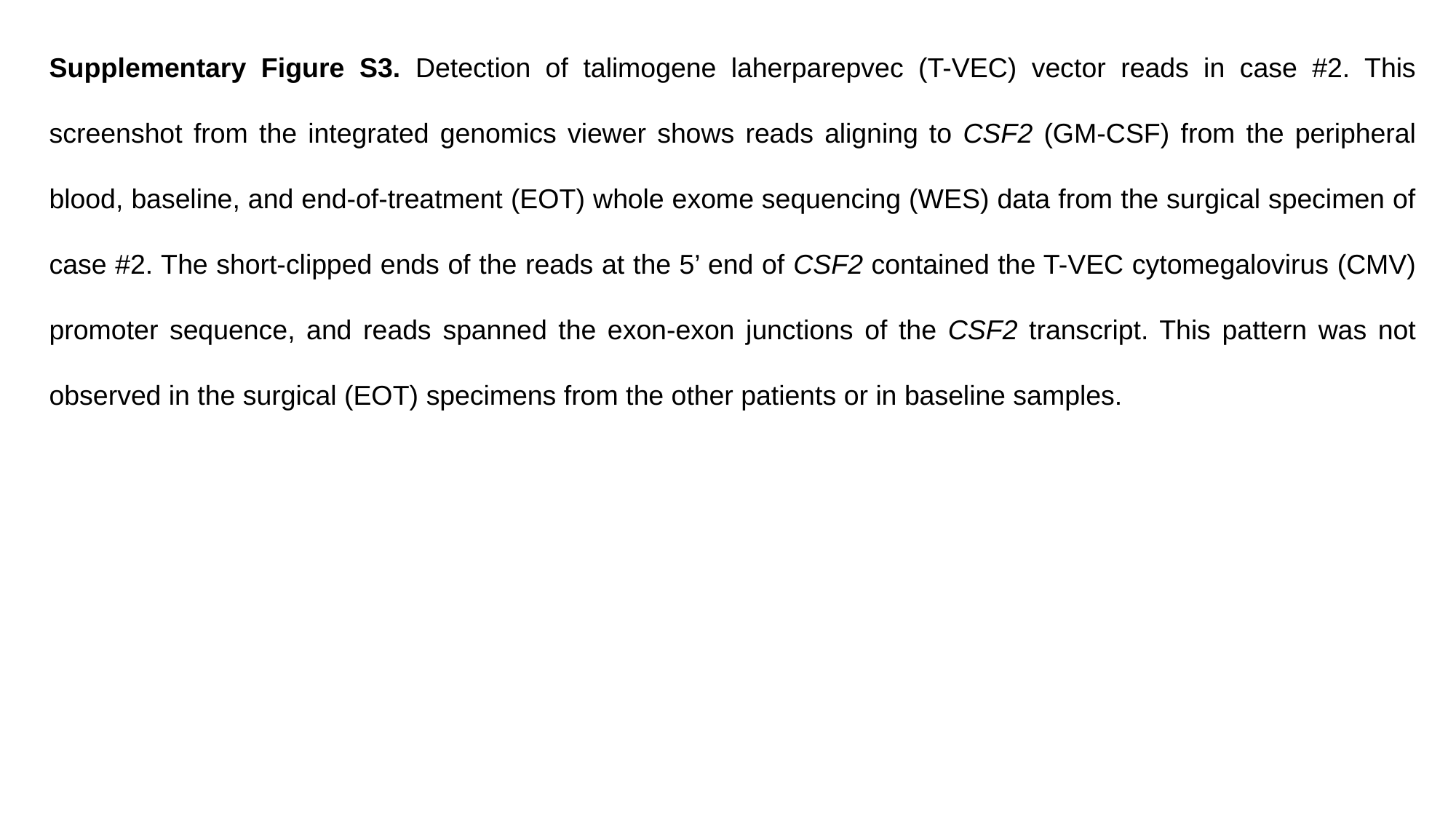

Supplementary Figure S3. Detection of talimogene laherparepvec (T-VEC) vector reads in case #2. This screenshot from the integrated genomics viewer shows reads aligning to CSF2 (GM-CSF) from the peripheral blood, baseline, and end-of-treatment (EOT) whole exome sequencing (WES) data from the surgical specimen of case #2. The short-clipped ends of the reads at the 5’ end of CSF2 contained the T-VEC cytomegalovirus (CMV) promoter sequence, and reads spanned the exon-exon junctions of the CSF2 transcript. This pattern was not observed in the surgical (EOT) specimens from the other patients or in baseline samples.
