# Supplementary material for: A Pilot Study of Neoadjuvant Nivolumab, Ipilimumab, and Intralesional Oncolytic Virotherapy for HER2-negative Breast Cancer
Source: Cancer Res Commun. 2023 Aug 23;3(8):1628–37. doi: 10.1158/2767-9764.CRC-23-0145 (PMC10445661; doi:10.1158/2767-9764.CRC-23-0145)
Supplement: Supplementary Table S1 — Representativeness of Study Participants [file crc-23-0145-s04.docx]

| **Supplementary Table S1. Representativeness of Study Participants** | |
| --- | --- |
| Cancer type(s)/subtype(s)/stage(s)/condition | Early stage triple negative breast cancer (TNBC) and hormone receptor positive, HER2-negative breast cancer. |
| Considerations related to: | |
| Sex | Breast cancer is a predominantly female disease and is rare in men. Male breast cancer represents only between 0.5 and 1% of all breast cancers diagnosed each year. TNBC incidence is around 10-15% among all breast cancer subtypes in the overall largely female population, but its incidence in males is lower (4%) when compared to the other subtypes. Hormone receptor positive, HER2-negative breast cancer incidence is around 60-70% among all breast cancer subtypes. |
| Age | The median age at the time of all breast cancer diagnoses is around 62. |
| Race/ethnicity | In the USA from 2014 to 2018, the overall breast cancer incidence rate was 127.1 cases per 100,000 among Black women and 132.5 cases per 100,000 among White women. |
| Geography | In the US, about 287,850 new cases of invasive breast cancer were diagnosed in 2021 and about 43,250 women die from breast cancer. |
| Other considerations | In large, randomized breast immunotherapy trials in which race/ethnicity are reported, the percentage of Black population is well below 13.4%, which is the US population average of Black individuals. Underrepresentation in trials limits evaluation of the impact of racial/ethnic- or ancestry-based differences in efficacy and toxicity.  The breast cancer death rate among Black women surpassed that of White women in the mid-1980s and continued to increase until the mid-1990s after which it started to decline in parallel in both groups. However, the 5-year relative survival rate for all subtypes of breast cancer diagnosed during 2011 through 2017 was still lower, 82% among Black women versus 92% among White women. |
| Overall representativeness of this study | Our study only included 6 subjects, but the age range was representative of the average for breast cancer (median age 55, range 40-72).  Of the 6 subjects, 3 were of non-White ethnicity including one Asian and one Black, so it included a fair representation of minorities.  As breast cancer in rare in men, no male TNBC patients were recruited, and were excluded from the study. |
