# Supplementary material for: A Pilot Study of Neoadjuvant Nivolumab, Ipilimumab, and Intralesional Oncolytic Virotherapy for HER2-negative Breast Cancer
Source: Cancer Res Commun. 2023 Aug 23;3(8):1628–37. doi: 10.1158/2767-9764.CRC-23-0145 (PMC10445661; doi:10.1158/2767-9764.CRC-23-0145)
Supplement: Supplementary Table S2 — Summary of antibodies, antigen retrieval conditions, incubation conditions, and opal dye labeling for multiplex immunofluorescence experiments [file crc-23-0145-s05.docx]

**Supplementary Table S2.** Summary of antibodies, antigen retrieval conditions, incubation conditions, and opal dye labeling for multiplex immunofluorescence experiments.

| Antibody | Vendor | Cat | Clone | Antibody Dilution | Antigen retrieval | Incubation time | Opal dye |
| --- | --- | --- | --- | --- | --- | --- | --- |
| IgG4 | Millipore | 411492 | HP6025 | 1:200 | ER2 20min | 30 mins | opal 620 |
| CD8 | DAKO | M7103 | C8/144B | 1:100 | ER2 20min | 30 mins | opal 480 |
| PD-1 | Cell Marque | NAT105 315m-96 | NAT105 | 1:50 | ER2 30min | 30 mins | opal 570 |
| PDL-1 | Spring/ Abcam | M4420/ ab228462 | SP142 | 1:100 | ER2 30min | 30 mins | opal 520 |
| CK7 | DAKO | M7018 | OV-TL | 1:200 | ER1 20min | 30 mins | opal 690 |
