# Supplementary material for: A Pilot Study of Neoadjuvant Nivolumab, Ipilimumab, and Intralesional Oncolytic Virotherapy for HER2-negative Breast Cancer
Source: Cancer Res Commun. 2023 Aug 23;3(8):1628–37. doi: 10.1158/2767-9764.CRC-23-0145 (PMC10445661; doi:10.1158/2767-9764.CRC-23-0145)
Supplement: Supplementary Table S3 — Individual details of baseline patient characteristics [file crc-23-0145-s06.docx]

**Supplementary Table S3.** Individual details of baseline patient characteristics.

| **Case #** | **Age (years)** | **Breast cancer subtype** | **Prior therapies** | **Recurrence after prior therapy** | **Lymph node status** |
| --- | --- | --- | --- | --- | --- |
| 1 | 40 | Triple negative | Chemotherapy with cyclophosphamide/doxorubicin and paclitaxel | No | Positive |
| 2 | 70 | Hormone receptor positive | Chemotherapy with gemcitabine and carboplatin | Yes | Negative |
| 3 | 47 | Triple negative | Chemotherapy with carboplatin and docetaxel | Yes | Negative |
| 4 | 57 | Triple negative | Chemotherapy with carboplatin and docetaxel | No | Positive |
| 5 | 72 | Hormone receptor positive | None | Yes | Positive |
| 6 | 52 | Triple negative | None | No | Positive |
