# Supplementary material for: A Pilot Study of Neoadjuvant Nivolumab, Ipilimumab, and Intralesional Oncolytic Virotherapy for HER2-negative Breast Cancer
Source: Cancer Res Commun. 2023 Aug 23;3(8):1628–37. doi: 10.1158/2767-9764.CRC-23-0145 (PMC10445661; doi:10.1158/2767-9764.CRC-23-0145)
Supplement: Supplementary Table S4 — Biopsy samples for analyses [file crc-23-0145-s07.docx]

**Supplementary Table S4.** Biopsy samples for analyses.

| Case # | Age (years) | Breast cancer subtype | Baseline biopsy | | | Resection biopsy | | |
| --- | --- | --- | --- | --- | --- | --- | --- | --- |
|  |  |  | Codex | WES | RNA | Codex | WES | RNA |
| 1 | 40 | Triple negative |  |  |  | X | X | X |
| 2 | 70 | Hormone receptor positive | X | X | X |  | X | X |
| 3 | 47 | Triple negative | X | X | X | X | X | X |
| 4 | 57 | Triple negative | X | X | X | X | X (2) | X (2) |
| 5 | 72 | Hormone receptor positive | X | X |  | X | X | X |
| 6 | 52 | Triple negative | X |  |  | X | X | X |
